# Principles, challenges, and advances in ribosome profiling: from bulk to low-input and single-cell analysis

**DOI:** 10.1007/s44307-023-00006-4

**Published:** 2023-12-01

**Authors:** Qiuyi Wang, Yuanhui Mao

**Affiliations:** 1 Bone Marrow Transplantation Center, the First Affiliated Hospital, School of Medicine, Zhejiang University, Hangzhou, China; 2 Liangzhu Laboratory, School of Medicine, Zhejiang University, Hangzhou, China

**Keywords:** Ribosome profiling, Ribo-seq quality, Dual-ligation, Template-switch

## Abstract

Ribosome profiling has revolutionized our understanding of gene expression regulation by providing a snapshot of global translation in vivo. This powerful technique enables the investigation of the dynamics of translation initiation, elongation, and termination, and has provided insights into the regulation of protein synthesis under various conditions. Despite its widespread adoption, challenges persist in obtaining high-quality ribosome profiling data. In this review, we discuss the fundamental principles of ribosome profiling and related methodologies, including selective ribosome profiling and translation complex profiling. We also delve into quality control to assess the reliability of ribosome profiling datasets, and the efforts to improve data quality by modifying the standard procedures. Additionally, we highlight recent advancements in ribosome profiling that enable the transition from bulk to low-input and single-cell applications. Single-cell ribosome profiling has emerged as a crucial tool for exploring translation heterogeneity within specific cell populations. However, the challenges of capturing mRNAs efficiently and the sparse nature of footprint reads in single-cell ribosome profiling present ongoing obstacles. The need to refine ribosome profiling techniques remains, especially when used at the single-cell level.

## Introduction

The ribosome is a molecular machine that translates the nucleotide sequence of messenger RNAs (mRNAs) into the amino acid sequence of proteins. mRNA translation is a pivotal process in gene expression and consumes a substantial amount of cellular energy (Liu et al. 2016; Lahtvee et al. 2017).

The translation of mRNA typically begins with the recruitment of the preinitiation complex, comprising the ribosomal small subunit, a ternary complex, and initiation factors, to the mRNAs. In prokaryotes, the preinitiation complex is loaded onto the initiation site, usually through a Shine-Dalgarno (SD) sequence located 8–10 nucleotides (nt) upstream of the initiation site (Rodnina 2018). In contrast, the recruitment of eukaryotic ribosomes is more intricate and appears to be a rate-limiting step in translation (Hinnebusch 2014; Merrick and Pavitt 2018). In eukaryotes, the preinitiation complex is recruited to the 5’ end of mRNA by recognizing the 5’ m^7^G cap through the eukaryotic initiation factor eIF4E. The preinitiation complex then scans the 5’ untranslated region (5’ UTR) until an initiation site is recognized. A commonly known scanning model suggests that the first AUG encountered by the initiation complex serves as the initiation site. However, the fidelity of initiation site selection often involves intricate interactions between initiation factors and *cis* elements (Kozak 2005; Hinnebusch 2011, 2017; Llácer et al. 2018; Brito Querido et al. 2020; Gu et al. 2021; She et al. 2023). It is not uncommon for the initiation complex to bypass several AUG triplets in the 5’ UTR through a mechanism known as leaky scanning (Kozak 1999; Dever et al. 2023). Furthermore, other AUG-like triplets in the 5’ UTR such as CUG, GUG, and UUG can also function as initiation codons in specific contexts, adding an additional layer of regulation to initiation site selection (Starck et al. 2012; Hinnebusch et al. 2016).

Once the initiation site is recognized, most initiation factors are released, and the ribosomal large subunit joins the initiation complex. Interestingly, while the scanning is rapid, the formation of ribosomes at the initiation site is time-consuming, potentially serving as a checkpoint for monitoring the fidelity of the initiation site and translation reading frame (Wang et al. 2019b, 2020, 2022; Lapointe et al. 2022; Mao et al. 2023). Upon the release of the initiation factor eIF5B, the initiation ribosome proceeds to the elongation cycle (Wang et al. 2019b). During elongation, the ribosome decodes nucleotide triplets (codon) in a sequential manner (Dever and Green 2012). The eukaryotic elongation factor eEF1 delivers amino acid-charged transfer RNAs (tRNAs) to the ribosome A site, and eEF2 catalyzes ribosome translocation. The rate and fidelity of decoding appear to be influenced by codon usage and the surrounding context (Liu et al. 2021). For example, non-optimal codons are translated more slowly and with lower accuracy compared to optimal codons due to a relative shortage of cognate tRNAs (Mordret et al. 2019). The resulting rhythm of translation elongation is critical for co-translational protein folding (Zhou et al. 2013; Yu et al. 2015) and mRNA stability (Presnyak et al. 2015; Hanson and Coller 2018). Interestingly, translation initiation can also be regulated by the elongation rate through a feedback pathway (Lyu et al. 2021).

When the ribosome reaches a stop codon, translation termination is triggered by termination factors (Hellen 2018), leading to the release of newly synthesized proteins and the recycling of ribosomes. Similar to initiation site selection, the fidelity of stop codon recognition is controlled by both *cis* elements and *trans* regulatory factors (Wangen and Green 2020). Intriguingly, other cognate tRNAs may also occasionally enter the ribosome A site at the stop codon, competing with the termination complex eRF1-eRF3 (Lawson et al. 2021). Mischarging of near cognate tRNAs at the stop codon can lead to ribosome readthrough, resulting in a C-terminal extension of the protein that can significantly impact mRNA and protein stability (Müller et al. 2023).

**Fig. 1:**
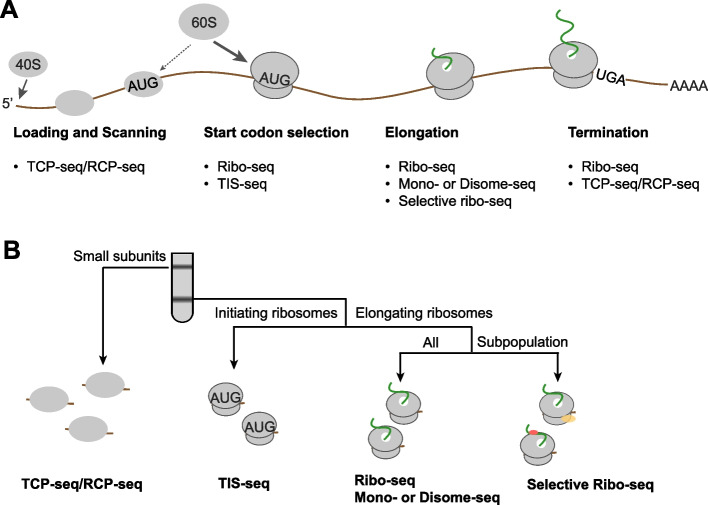
Translational control and ribosome profiling. **A** A schematic diagram illustrating the main steps of mRNA translation and the high-throughput sequencing methods involved. **B** The relationship between ribosome profiling and other high throughput sequencing methods

Despite extensive research on translation regulation, numerous fundamental questions remain unanswered. For instance, the precise roles of initiation factors and trans regulatory factors in guiding preinitiation complex loading, scanning and start site selection are not fully understood. Moreover, there is ongoing debate regarding the correlation between mRNA stability and translation, as conflicting findings have been reported (Dave et al. 2023). Emerging technologies such as single-molecule fluorescence (Prabhakar et al. 2019), massively parallel reporter assays (Jia et al. 2020; Kesner et al. 2023) and multi-omics approaches have advanced our understanding of translation regulation. Ribosome profiling, also known as Ribo-seq, is a technology that provides a snapshot of global translation in cells by sequencing the RNA fragments protected by translating ribosomes (Ingolia et al. 2009; Brar and Weissman 2015). Since its first application in yeast translation in 2009, ribosome profiling has been adapted to various types and widely used in numerous studies (Fig. 1). Over the past 15 years, there has been a great effort to improve the quality of ribosome profiling, however, obtaining high-quality Ribo-seq data is still technically challenging. In this review, we will summarize the applications of ribosome profiling and related high-throughput sequencing technologies. Furthermore, we will discuss quality control of ribosome profiling and highlight the procedures that determine its quality.

## Ribosome profiling, selective ribosome profiling and translation complex profiling

**Fig. 2:**
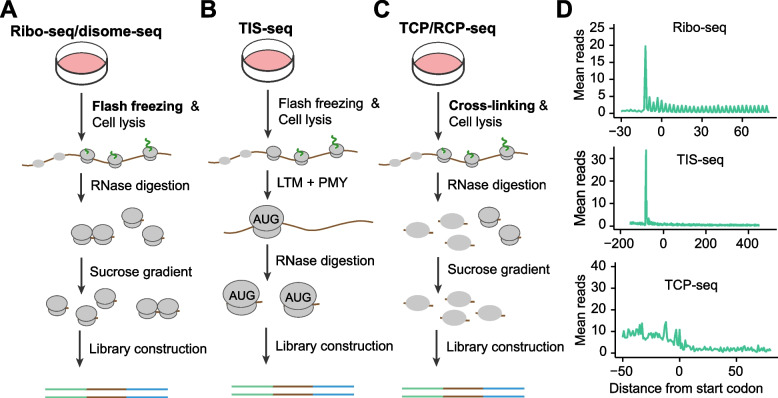
Procedures of ribosome profiling and related high throughput sequencing methods. **A** Ribosome profiling and Disome-seq. Cells were subjected to flash freezing and cell lysis. The lysates were digested by RNase (e.g. RNase I), and the digested products were fractionated by a sucrose gradient, separating RNA fragments into different fractions based on the number of ribosomes. The monosome fraction was collected to construct a ribosome profiling library, and the disome fraction was collected for disome-seq. **B** Translation initiation site sequencing (TIS-seq). The procedure is similar to standard ribosome profiling, expect for the use of lactimidomycin (LTM) and puromycin (PMY). LTM specifically binds to the initiating ribosome, stabilizing the ribosome at the start codon, while PMY releases the nascent chain and dissociates the elongating ribosomes. **C** TCP/RCP-seq. In contrast to ribosome profiling, the ribosome subunits were immobilized on mRNAs through cross-linking. The RNase digestion products were separated by a sucrose gradient, and the fraction containing small subunits was collected for library construction. **D** Representative examples of aggregation plots for ribosome profiling, TIS-seq and TCP-seq. The data from GSE176058 and GSE159210 were used

Ribosome profiling hinges upon the principle that ribosomes shield specific regions of messenger RNA (mRNA) from ribonuclease digestion. This methodology entails sequencing the residual RNA fragments following RNase treatment, denoted as ribosome-protected RNA fragments or ribosome footprints, to precisely determine the positions of translating ribosomes on mRNAs (Fig. 2A and D). In general, the count of footprints directly indicates the abundance of actively translating ribosomes on the mRNA. Therefore, ribosome profiling has been widely used to quantify translation activity, where a reduced footprint count indicates a decrease in translation activity and vice versa (Xiao et al. 2016). For instance, under environmental perturbations, such as nutrient and hyperosmotic stress (Darnell et al. 2018; Wu et al. 2019; Jobava et al. 2021), cells can rapidly respond to stress by suspending global translation and increasing the translation of stress response genes including *ATF4*, *CHOP* and *GADD34*. In addition, it is worth noting that footprint reads within CDs are affected by both initiation and elongation rate. Therefore, differential changes of translation levels can be confounded by the accumulation of ribosomes within CDS such as ribosome pausing induced by stress. While it is possible to remove outliers of footprint reads from CDS, those outlier-trimmed methods remain to be evaluated.

Despite the prevalent utilization of ribosome profiling for mRNA translation differential analysis, it is widely employed for discovering small open reading frames (smORFs). Numerous computational tools by leveraging ribosome profiling data have been developed to identify smORFs across diverse species, yielding the number of smORFs ranging from tens of thousands to several million (Ingolia et al. 2014; Calviello et al. 2016; Olexiouk et al. 2016; McGillivray et al. 2018; Clauwaert et al. 2019; Wang et al. 2019a; Martinez et al. 2020; Mudge et al. 2021, 2022; Sandmann et al. 2023). Recently, a study based on super high-quality ribosome profiling datasets reported a notably lower number of smORFs (~ 7,000 smORFs) differentially expressed in different human tissues (Chothani et al. 2022). Notably, smORFs identification is sensitive to the quality of ribosome profiling (Lei et al. 2023). To date, the precise number of smORFs in various species remains elusive (Mudge et al. 2022), primarily due to the stringent requirements for smORFs identification, necessitating high-quality ribosome profiling.

In contrast to capturing all translating ribosomes on mRNAs, standard ribosome profiling has been adapted to selectively enrich ribosomes bound by co-translational factors, referred to as selective ribosome profiling. As an example, co-translational folding of nascent peptides, a pivotal process for maintaining protein homeostasis, is facilitated by the ribosome associated complex (RAC) (Zhang et al. 2020; Kišonaitė et al. 2023). The Hsp70 family member HSP70 (HSPA1A/B in human and Ssb1/Ssb2 in yeast) targets ribosomes through the RAC complex, thus aiding the co-translational folding of nascent peptides (Hanebuth et al. 2016; Chen et al. 2022). To investigate the function of the Ssb protein, previous studies performed ribosome profiling combined with a procedure to selectively isolate Ssb-associated ribosomes (Oh et al. 2011; Döring et al. 2017; Shiber et al. 2018; Stein et al. 2019). This approach revealed that Ssb shields hydrophobic patches within interaction domains, thus safeguarding nascent peptides against non-productive interactions and misfolding. Another example comes from the study on neuromuscular disease spinal muscular atrophy (SMA), a neuromuscular disease associated with the depletion of the survival motor neuron (SMN) protein. Employing selective ribosome profiling to enrich SMN-bound ribosomes, a previous study found that the SMN protein preferentially associates with ribosomes positioned within the first five codons of a subset of mRNAs linked to SMA pathogenesis, thereby playing a pivotal role in the pathogenic cascade of SMA (Lauria et al. 2020). In a previous study on the Huntington disease (Eshraghi et al. 2021), a debilitative autosomal-dominant brain disorder characterized by the loss of language and behavioral abilities, the Huntingtin protein (mHTT) can promote ribosome pausing on specific mRNAs, subsequently impeding ribosome translocating during elongation.

Notably, selective ribosome profiling can also be performed by focusing on specific subsets of ribosomes on mRNAs. Examples of these specialized methodologies include MitoRiboSeq for the enrichment of mitochondrial ribosomes (Morscher et al. 2018; Li et al. 2021), TIS-seq for the specific capture of initiation ribosomes at translation start sites (Lee et al. 2012; Gao et al. 2015; Zhang et al. 2017; Eisenberg et al. 2020) (Fig. 2B and D), di-/mono-ribosome profiling for the analysis of di-/mono- ribosomes on mRNAs (Biever et al. 2020; Meydan and Guydosh 2020; Zhao et al. 2021; Ferguson et al. 2023). As an example, strong ribosomal pausing can lead to the formation of di-ribosomes on mRNAs, which can inhibit translation elongation and trigger the ribosome-associated protein quality control pathway (Ikeuchi et al. 2019). Because di-ribosomes are resistant to RNase I digestion, they are often excluded from the standard ribosome profiling when a sucrose cushion is used for the isolation the monosome generated by RNase I digestion. Therefore, di-ribosome profiling, using polysome profiling to separate di-ribosome from mono-ribosome after RNase I digestion, can largely enrich di-ribosomes, which revealed a distinct ribosome pausing during elongation and in the region before the stop codon (Meydan and Guydosh 2020; Zhao et al. 2021). In addition, the selective ribosome profiling can be performed in vivo (Gonzalez et al. 2014; Doroudgar et al. 2019). For instance, the ribosomal large subunit RPL22 was genetically fused with a hemagglutinin (HA) tag (Doroudgar et al. 2019). The expression of tagged RPL22 was specifically induced in the mouse heart using a heart specific Cre recombinase-expressing system, resulting in the synthesis of HA-RPL22-tagged ribosomes. By purifying tagged ribosome-associated mRNAs and performing ribosome profiling, a previous study revealed a potential role of the upstream open reading frame (uORF) in cardiac metabolism through the regulation of the main open reading frame (mORF) translation.

In addition to standard and selective ribosome profiling, another prevalent approach involves the capture of ribosome subunits rather than intact 80S ribosomes on mRNAs (Archer et al. 2016; Giess et al. 2020; Wagner et al. 2020). The ribosomal subunits are often immobilized through crosslinking methods, which are subsequently separated using a sucrose gradient (Fig. 2C and D). The RNA fragments covered by ribosomal subunits are then extracted and subjected to high-throughput sequencing. The method is generally referred to as translation complex profiling (TCP-seq) or ribosome complex profiling (RCP-seq). Unlike standard ribosome profiling, TCP/RCP-seq monitors ribosomal subunits, providing a unique opportunity to investigate regulations during translation initiation. Notably, TCP/RCP-seq can also be adapted to selectively enrich ribosomal subunits associated with initiation factors, a variant termed selective TCP/RCP-seq (Wagner et al. 2020, 2022). Through selective TCP-seq, previous studies have revealed molecular details of the assembly of ribosome subunits and associated initiation factors including eIF2 and eIF3, at various stages of translation initiation (Wagner et al. 2020).

## Quality control of ribosome profiling

Quality control is a crucial aspect of assessing the reliability of a ribosome profiling dataset, due to the susceptibility of this method to issues such as low quality and sequence bias. Several steps can be employed to evaluate a ribosome profiling experiment.

### Footprint length

**Fig. 3:**
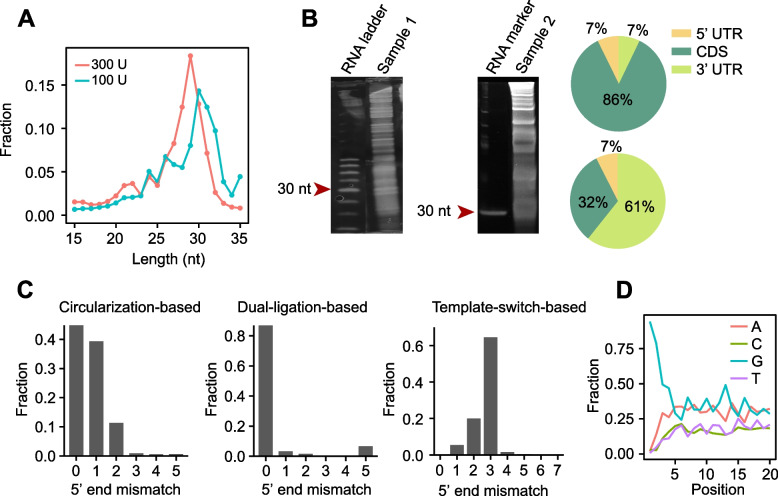
Quality control of ribosome profiling. **A** Line plots show the distribution of footprint length using cell lysates treated with different RNase I concentrations (AM2295, 100 U versus 300 U), suggesting that a lower RNase I concentration may reduce RNA digestion efficiency. **B** Quality check of RNase I digestion. After RNase I digestion, RNA fragments were separated on a 15% denatured PAGE gel, which was then stained with SYBR Gold. The RNA ladder (left) or marker (30 nt) is shown in lane 1 of each panel. Compared to the left panel (100 U RNase I, AM2295), the fragments at approximately 30 nt were relatively weak when an extremely low concentration of RNase I (10 U RNase I, AM2295) was used. When RNAs are efficiently digested, the majority of fragments approximately 30 nt are aligned to CDS, whereas the fragments are contaminated with RNA fragments from the 3’ UTR when the digestion is inefficient (right pie chart). **C** Bar plots show the accuracy of the 5’ end of the footprint when different library construction methods were used. Circularization-based methods have ~ 50% of reads showing additional non-templated nucleotides at the 5’ end of footprint reads (mismatches > 0, left panel, data from SRR7241912 were used). Compared to circularization-based methods, dual-ligation methods significantly improve 5’ end accuracy, with < 5% footprint reads having mismatches > 0 (data from SRR14824510 were used). Template-switch-based methods add a variable number of non-templated nucleotides to the 5’ end of cDNA (right panel, unpublished data). **D** The G preference of template-switch methods. Position refers to the positions from the 5’ end of footprint reads

The typical length of ribosome footprints in most species is approximately 30 nucleotides (nt), representing the region covered by a translating ribosome (Ingolia et al. 2009). However, the footprint length can vary, ranging from 20 to 31 nt. By analyzing various ribosome profiling datasets, we found that the 5’ end of footprints, the left boundary protected by ribosomes, is relatively fixed, whereas the 3’ end exhibits flexibility, leading to a variation in footprint length (Mao et al. 2023). Footprint length is notably influenced by RNase digestion (Fig. 3A) (Gerashchenko and Gladyshev 2017; Douka et al. 2021). Inefficient digestion tends to yield longer footprints, which compromises the quality of ribosome profiling. However, variation in footprint length could indicate ribosome heterogeneity. For example, during amino acid starvation, an increase in short footprints of approximately 21 nt indicates paused ribosomes with empty A sites due to the absence of cognate charged tRNAs (Wu et al. 2019).

### Reads in coding region

A vast majority (> 80%) of footprint reads aligned to mRNAs are typically found within the coding region (CDS) (Fig. 3B). A notably lower percentage of CDS reads usually indicates a higher level of contamination and lower data quality. In addition, it is worth investigating whether reads aligned to non-coding regions or non-coding RNAs represent active translation or are background noise (Couso and Patraquim 2017; Wright et al. 2022; Mao and Qian 2023). For instance, a typical ribosome profiling dataset in human HEK293 cells exhibits 5–10% footprint reads in the 5’ UTR, implying potential active translation in the 5’ UTR of certain mRNAs (Fig. 3B). Indeed, it has been well accepted that alternative translation initiation can occur in the 5’ UTR when an initiation complex scans along the 5’ UTR and encounters an optimal initiation context upstream of the annotated start codon (Medenbach et al. 2011; Dever et al. 2020; Orr et al. 2020). In addition, footprint reads in the 3’ UTR may indicate stop codon readthrough (Dunn et al. 2013; Arribere et al. 2016) or reinitiation (Young et al. 2015a, 2015b; Shu et al. 2022) after translation termination. However, given that readthrough and reinitiation are relatively rare on most mRNAs and that there are many RNA binding proteins in the 3’ UTR, most sequencing reads in the 3’ UTR are likely contaminated by background noise and should be carefully evaluated. Identifying non-canonical translation events in non-coding regions and non-coding RNAs is of special interest (Chen et al. 2020; Prensner et al. 2021). For example, alternative initiation is common in stressed cells (Hinnebusch et al. 2016; Young and Wek 2016) and in many types of cancer cells (Wang et al. 1996; Sendoel et al. 2017; Xu et al. 2019; Huang et al. 2021), which implies an important role for alternative initiation in stress responses and tumorigenesis.

### In-frame rate

The in-frame rate (IFR) denotes the fraction of footprint reads aligned to the correct reading frame of the CDS. IFR is a critical quality control metric for ribosome profiling, particularly in studies focusing on ribosome occupancy at individual codons to investigate ribosome pausing or frameshifting. A lower IFR in a ribosome profiling dataset indicates lower data quality and potential contamination from non-specific mRNA fragments during library preparation. Such contaminants can significantly affect the estimation of translation activity, typically calculated based on all footprint reads on mRNAs. In addition, IFR is vital for identifying new ORFs, as most computational tools use IFR to discriminate ORFs from non-coding regions (Bazzini et al. 2014; Mackowiak et al. 2015; Calviello et al. 2016). Currently, most ribosome profiling experiments yield datasets with an IFR of approximately 60–70%. Recently, we developed a novel library construction method for ribosome profiling, achieving an IFR up to 90% in HEK293 cells (Mao et al. 2023). This high-resolution dataset revealed prevalent ribosome frameshifting during the early stage of elongation. Interestingly, the frameshifting rate on individual codons seems to be associated with codon optimality, with non-optimal codons (lacking cognate tRNAs) showing a significantly higher frameshifting rate than optimal codons. It is important to note that IFR in most ribosome profiling datasets is length-dependent. Generally, footprint reads with length of approximately 28 nt have the highest IFR, often reaching 80% or higher. IFR decreases dramatically for reads shorter than 28 nt, which should be carefully evaluated when calculating ribosome occupancy using short reads. Notably, the IFR is mainly affected by the accuracy of the 5’ end of footprint reads, which is also influenced by library construction methods (Fig. 3C and D, discussed later).

### Aggregation plot

An aggregation plot displays an averaged ribosome density along the CDS, which is commonly used to examine ribosome pausing at specific codons. However, an abnormal increase in ribosome density near the start codon can indicate potential concerns with data quality, probably resulting from ribosome movement during cell harvesting (Sharma et al. 2021).

## Challenges in ribosome profiling and methodology

Ribosome profiling, although widely embraced, poses technical challenges in generating high-quality datasets. This complexity arises from the intricate nature of the ribosome profiling procedure, which encompasses numerous critical steps, each harboring distinct technical intricacies that can adversely affect the integrity of the data.

### Cell harvesting and lysis

The initial step involves stabilizing ribosomes on mRNA molecules before cell lysis. To this end, cells are subjected to flash freezing to arrest translation activity (McGlincy and Ingolia 2017). However, a potential concern arises during cell harvesting and lysis in cold conditions, as mRNA translation is sensitive to stress including cold (Knight et al. 2015). Low temperature before cell harvesting may alter the translation landscape (Zhang et al. 2018). Therefore, harvesting cells as soon as possible may improve the quality of ribosome profiling. Another common method to arrest translation is pre-incubating cells with cycloheximide. Cycloheximide blocks translation elongation by binding to ribosomes and inhibiting eEF2-mediated translocation (Ennis and Lubin 1964; Baliga et al. 1969; Schneider-Poetsch et al. 2010). Intriguingly, previous studies have reported that cycloheximide allows one complete translocation cycle before halting further elongation (Schneider-Poetsch et al. 2010), raising questions about its ability to immobilize ribosomes in situ and thus complicating ribosome occupancy at individual codons. Systematic analysis of ribosome profiling datasets in yeast with and without cycloheximide has indeed revealed biases in ribosome occupancy introduced by cycloheximide (Lareau et al. 2014; Hussmann et al. 2015). In addition, cycloheximide-induced translation elongation inhibition can induce stress responses, which may perturb the global translation landscape (Santos et al. 2019). Although the use of cycloheximide in mammalian cells remains controversial (Sharma et al. 2021), it warrants particular attention when pre-incubating cells with cycloheximide.

### Polysome profiling

Polysome profiling is a technique that segregates translated mRNAs on a sucrose gradient based on the number of bound ribosomes (Chassé et al. 2017; Pringle et al. 2019). Following ultracentrifugation of the cell lysate on a 15–40% sucrose gradient, actively translated mRNAs can be separated into different fractions according to ribosome count. These mRNA fractions are subsequently collected using spectrophotometric analysis at A254. Polysome profiling effectively enriches ribosome-bound mRNAs, resulting in a significant reduction in non-coding RNA content, improved RNase digestion efficiency, and, consequently, enhanced ribosome profiling data quality. However, polysome profiling requires a substantial amount of RNA and may not be suitable for low-input samples (Liang et al. 2018). In addition, a fraction analyzer is needed for polysome profiling, which may not be available to all research groups (Sobhany and Stanley 2021). Consequently, many ribosome profiling experiments opt to omit this step. In an alternative approach, chemical labeling techniques such as puromycin labeling have been developed to enrich ribosome-bound mRNAs (Schmidt et al. 2009; Kandala et al. 2019; Hadidi et al. 2023). Puromycin, an aminonucleoside antibiotic, binds to ribosomes and nascent peptide chains, offering a means to quantify protein synthesis (Semenkov et al. 1992; Starck and Roberts 2002). Previous studies have introduced methods such as RiboLace, which utilize puromycin-containing molecules to isolate active ribosomes. (Clamer et al. 2018).

### RNA digestion

Efficient RNA digestion plays a pivotal role in achieving high-quality ribosome profiling (Li et al. 2023). RNase I is the most commonly used RNase for ribosome profiling. Digestion is usually completed at room temperature (McGlincy and Ingolia 2017). However, a recent study suggested that overnight digestion on ice could enhance digestion efficiency (Douka et al. 2021). It is important to note that RNase I digests ribosomal RNAs (rRNA), resulting in significant rRNA contamination in ribosome profiling libraries (Meador et al. 1990). rRNA contamination consumes a vast majority of sequencing reads, significantly reducing the number of reads aligned to mRNAs (low to 5% of total reads). It is challenging to remove rRNA contamination from ribosome profiling libraries (Thompson et al. 2020; Zinshteyn et al. 2020), therefore, other RNases have been explored to mitigate rRNA contamination (Gerashchenko and Gladyshev 2017; Hwang and Buskirk 2017), including micrococcal nuclease (MNase) (VanInsberghe et al. 2021), RNase A (Simsek et al. 2017), and RNase T1 (Liu et al. 2018; Gerashchenko 2021). However, these RNases often exhibit RNA sequence preferences in digestion, introducing additional biases to the calculation of ribosome occupancy at individual codons (VanInsberghe et al. 2021). Recently, a study indicated that the nuclease P1 may be a promising alternative with the potential to digest mRNAs without obvious sequence bias (Ferguson et al. 2023). In addition, nuclease P1 appears to have a preference for mRNA over rRNA compared to RNase I, resulting in a reduction of the rRNA fraction to less than 50% (Ferguson et al. 2023).

### Library construction

**Fig. 4:**
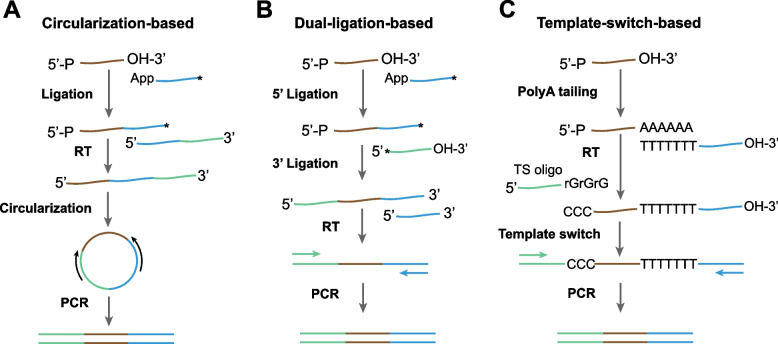
Library construction method for ribosome profiling. **A** Circularization-based, **B** Dual-ligation-based, and **C** Template-switch-based. RT: reverse transcription

Selecting an appropriate methodology for constructing ribosome profiling libraries is crucial. Initially, circularization-based methods were employed, involving RNA ligation and DNA circularization (Ingolia et al. 2009; McGlincy and Ingolia 2017). RNA fragments were first ligated to a 3’ adaptor, which served as priming sites for reverse transcription. After reverse transcription, the complementary DNA (cDNA) was circularized and then subjected to PCR amplification using the priming sites in the 3’ adaptor (Fig. 4A). The circularization-based method is time-consuming and inefficient, rendering it unsuitable for low-input RNA samples. Furthermore, reverse transcription introduces non-templated nucleotides at the 5’ end of cDNA, due to the non-templated addition of reverse transcriptase. Although non-templated nucleotides can be removed using computational methods, it is impossible to discriminate additional nucleotides if they can be aligned to the genome, which significantly reduces the 5’ end accuracy of footprint reads (Fig. 3C). To improve the efficiency of library construction, other methods such as the dual-ligation method have been employed (VanInsberghe et al. 2021). Dual-ligation methods, which ligate fragments to both 3’ and 5’ adaptors, offer higher efficiency and are suitable for single-cell ribosome profiling (Fig. 4B). The major concern of dual-ligation is the sequence preference of ligation, which potentially alter the relative ribosome densities at individual codons. To mitigate ligation bias, random barcodes have been introduced into the ligation primers (Lecanda et al. 2016; McGlincy and Ingolia 2017; Lama et al. 2019). Notably, random barcodes can also serve as unique molecular identifiers (UMI) to correct PCR amplification biases. In addition, dual-ligation methods are also prone to formation of dimers, a self-ligation between 5’ and 3’ adaptors. Dimmers consume a substantial fraction of adaptors and sequencing depth and thus must be eliminated from libraries through gel electrophoresis. Recently, another method based on the template-switch mechanism has been utilized for ribosome profiling library construction (Li et al. 2022, 2023; Xiong et al. 2022; Zhang et al. 2022; Zou et al. 2022). In this approach, RNA fragments are tagged with polyadenine tails and subjected to reverse transcription using Moloney murine leukemia virus (MMLV)-type reverse transcriptase with a switching property (Wulf et al. 2019). When reverse transcription reaches the 5’ end of RNA fragments, the reverse transcriptase tends to add three additional non-templated nucleotides (+ CCC) to the 3’ end of nascent cDNAs, which allows the reverse transcriptase to switch templates to the templated switched oligo (TSO) sequence (Fig. 4C). Because the amount of input RNAs for the template-switch method can be extremely low, this method has been widely used in single-cell RNA-seq library construction (Picelli et al. 2013, 2014). However, a few concerns should be considered when it is used for single-cell ribosome profiling. First, non-templated nucleotides at the 3’ end of cDNAs are generally variable, with a median value of 3 nucleotides (Fig. 3C). The variable 3’ end makes it challenging to infer the accurate 5’ end of footprints and thus the positions of ribosomes. Therefore, mathematical models such as random forest have been trained to predict ribosome positions, based on footprint reads around the stop codon, where ribosome positions are assumed to be accurate due to ribosome pausing at the stop codon (VanInsberghe et al. 2021). Additionally, template-switch methods exhibit a bias toward guanine, potentially enriching footprint reads with G at the 5’ ends and introducing biases that may impact ribosome occupancies at individual codons (Fig. 3D) (Tang et al. 2013; Meistertzheim et al. 2019).

## Advancements in ribosome profiling: transitioning from bulk to low-input and single-cell ribosome profiling

Ribosome profiling-based methodologies have significantly improved our capacity to monitor protein synthesis in vivo. Similar to RNA-seq, conventional bulk ribosome profiling falls short in detecting translation controls within specific cell populations. To scrutinize translation heterogeneity at the single-cell level, single-cell ribosome profiling based on the dual-ligation method was introduced (VanInsberghe et al. 2021). Single-cell ribosome profiling revealed distinct responses in translation activity and ribosome pausing throughout the cell cycle. Remarkably, ribosome pausing at codons encoding specific amino acids was evident only in certain cells, dependent on their cell cycle state. It is believed that depletion of single amino acids or cognate tRNAs leads to ribosome pausing at specific codons. However, most bulk ribosome profiling data in mammals have failed to establish a clear correlation between ribosome pausing and the corresponding tRNA or amino acid abundance. The heterogeneity of ribosome pausing revealed by single-cell ribosome profiling may elucidate the absence of such a correlation.

In contrast to the ligation-based library used in previous studies (VanInsberghe et al. 2021; Froberg et al. 2023), several recent studies have employed the template-switch method to construct low-input ribosome profiling libraries using only a few hundred cells (Li et al. 2022; Xiong et al. 2022; Zhang et al. 2022; Ozadam et al. 2023). By applying low-input ribosome profiling to cells during the early development of embryos, a stage characterized by more active mRNA translation than transcription, previous studies (Zhang et al. 2022; Ozadam et al. 2023)have revealed a remarkable alteration in the translation landscape within mammalian oocytes or embryos.

While both low-input and single-cell ribosome profiling offer insights into translation control at the single-cell level, cell subpopulations often need to be separated before ribosome profiling. However, cell sorting can introduce stress responses due to the sensitivity of translation, leading to rapid changes in the global translation landscape. Recently, a spatially resolved single-cell translatomics method termed RIBOMap was developed (Zeng et al. 2023). Using a tri-probe system that includes probes targeting rRNA and specific groups of mRNAs, and probe functions for DNA amplification, RIBOMap can detect and quantify ribosome-bound mRNA in situ. By applying RIBOMap in the mouse brain in parallel with spatial transcription analysis, a previous study (Zeng et al. 2023) revealed significantly differential translation regulation during oligodendrocyte maturation. Notably, RIBOMap is also able to monitor translation activity at the subcellular level, revealing translation control in specific localization within cells.

## Perspective

Over the past 15 years, ribosome profiling has evolved significantly, emerging as a versatile method for monitoring translation activity at various stages of protein synthesis and in diverse cell types, tissues and numerous human diseases (Lee et al. 2021; Ouspenskaia et al. 2022; Passarelli et al. 2022). Moreover, the ribosome profiling procedure has been streamlined, leading to the generation of high-quality datasets (Ferguson et al. 2023). Nevertheless, there persists a need for further refinement in ribosome profiling quality to address fundamental questions in translation control. For instance, only a small fraction of sequencing reads generated in most ribosome profiling experiments can be successfully aligned to mRNAs, typically ranging from 10 to 30%. Regardless of rRNA contamination, a significant proportion of reads are mapped to non-coding RNAs or non-coding regions of mRNAs, suggesting pervasive translation in certain non-coding regions (Orr et al. 2020; Wright et al. 2022; Mao and Qian 2023). Identifying such non-canonical translation within non-coding regions poses challenges, primarily owing to their typically lower translation levels than mRNAs. While computational tools play a crucial role in unveiling non-canonical translation events (Lei et al. 2023), improving the quality of ribosome profiling can substantially reduce background noise, thus increasing the sensitivity and specificity of the identification of non-canonical translation events.

**Fig. 5:**
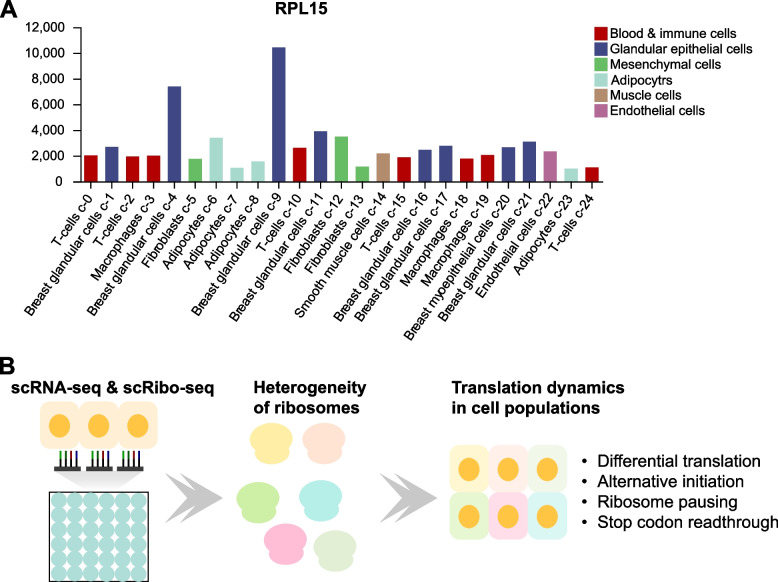
Ribosome heterogeneity and single-cell ribosome profiling. **A** Expression levels of ribosomal protein RPL15 across different cell types in the breast. Data from the Human Protein Atlas were used. **B** A schematic diagram illustrating ribosome heterogeneity and the diversity of translation regulation across cell types which can be revealed by a single-cell ribosome profiling (scRibo-seq) combined with a single-cell RNA-seq (scRNA-seq)

Another intriguing question pertains to single-cell and spatial ribosome profiling. Emerging evidence indicates significant variations in ribosome composition among different tissues and cell types (an example showing Fig. 5A) (Genuth and Barna 2018; Gay et al. 2022). Ribosomal heterogeneity plays a pivotal role in the specialized translation of specific groups of mRNAs which can be mediated by the diversity in ribosomes. Single-cell ribosome profile together with single-cell RNA-seq offers a powerful approach to investigate the regulation and function of specialized translation (Fig. 5B). Although single-cell ribosome profiling techniques are available, many of them suffer low efficiency. First, only a small fraction of mRNAs is captured, limiting the ability to detect the translation landscape in cell populations. Second, the unique reads in each cell are rather scarce. The extreme sparsity of footprint reads makes it challenging to calculate ribosome occupancy at individual codons. Theoretically, techniques such as RIBOMap have the potential to capture a higher number of mRNAs by increasing the number of probes. However, they lack information about ribosome positions, which limits their ability to detect the heterogeneity of ribosome occupancy in tissues. For example, uORF translation plays critical roles in translation regulation of downstream main ORFs (Dever et al. 2023). Whether there is heterogeneity in uORF translation in a cell-specific manner remains unclear. The development of high-quality single-cell ribosome profiling may enhance our comprehension of the heterogeneity of uORF translation.

## Data Availability

Not applicable.
